# A multicenter study on developing a prognostic model for severe fever with thrombocytopenia syndrome using machine learning

**DOI:** 10.3389/fmicb.2025.1557922

**Published:** 2025-03-19

**Authors:** Jian-She Xu, Kai Yang, Bin Quan, Jing Xie, Yi-Shan Zheng

**Affiliations:** ^1^School of Public Health, Nanjing Medical University, Nanjing, China; ^2^Department of Intensive Care Unit, The Second Hospital of Nanjing, Affiliated of Nanjing University of Chinese Medicine, Nanjing, China; ^3^Department of Infectious Disease, The First Affiliated Hospital of Wannan Medical College, Wuhu, China

**Keywords:** severe fever with thrombocytopenia syndrome, machine learning, Boruta algorithm, prognostic model, clinical validation

## Abstract

**Background:**

Severe Fever with Thrombocytopenia Syndrome (SFTS) is a disease caused by infection with the Severe Fever with Thrombocytopenia Syndrome virus (SFTSV), a novel Bunyavirus. Accurate prognostic assessment is crucial for developing individualized prevention and treatment strategies. However, machine learning prognostic models for SFTS are rare and need further improvement and clinical validation.

**Objective:**

This study aims to develop and validate an interpretable prognostic model based on machine learning (ML) methods to enhance the understanding of SFTS progression.

**Methods:**

This multicenter retrospective study analyzed patient data from two provinces in China. The derivation cohort included 292 patients treated at The Second Hospital of Nanjing from January 2022 to December 2023, with a 7:3 split for model training and internal validation. The external validation cohort consisted of 104 patients from The First Affiliated Hospital of Wannan Medical College during the same period. Twenty-four commonly available clinical features were selected, and the Boruta algorithm identified 12 candidate predictors, ranked by Z-scores, which were progressively incorporated into 10 machine learning models to develop prognostic models. Model performance was assessed using the area under the receiver-operating-characteristic curve (AUC), accuracy, recall, and F1 score. The clinical utility of the best-performing model was evaluated through decision curve analysis (DCA) based on net benefit. Robustness was tested with 10-fold cross-validation, and feature importance was explained using SHapley Additive exPlanation (SHAP) both globally and locally.

**Results:**

Among the 10 machine learning models, the XGBoost model demonstrated the best overall discriminatory ability. Considering both AUC index and feature simplicity, a final interpretable XGBoost model with 7 key features was constructed. The model showed high predictive accuracy for patient outcomes in both internal (AUC = 0.911, 95% CI: 0.842–0.967) and external validations (AUC = 0.891, 95% CI: 0.786–0.977). A clinical tool based on this model has been developed and implemented using the Streamlit framework.

**Conclusion:**

The interpretable XGBoost-based prognostic model for SFTS shows high predictive accuracy and has been translated into a clinical tool. The model's 7 key features serve as valuable indicators for early prognosis of SFTS, warranting close attention from healthcare professionals in clinical practice.

## Introduction

Severe Fever with Thrombocytopenia Syndrome (SFTS) is an acute infectious disease caused by the Severe Fever with Thrombocytopenia Syndrome virus (SFTSV), a member of the Bunyaviridae family, and is primarily transmitted through tick bites (Yu et al., 2011). SFTS has a high incidence in East Asia, with a fatality rate of up to 30%, particularly in China, Japan, and South Korea (Kim et al., 2013; Liu et al., 2014). The clinical characteristics of the disease include high fever, thrombocytopenia, leukopenia, and multi-organ dysfunction, with severe cases often resulting in death (Gai et al., 2012; Liu et al., 2014). Currently, there is no specific treatment available, making early identification of critically ill patients crucial for improving outcomes (Wang et al., 2021). Clinically, there is a lack of accurate and reliable prognostic models to predict disease progression and patient outcomes (Jia et al., 2017; Liu et al., 2023). As a heterogeneous disease, the assessment of disease severity and prognosis in SFTS patients relies on various clinical and laboratory indicators.

Routine blood tests (RBT) play a critical role in the diagnosis, prognosis, and follow-up of many diseases (Huyut, 2023; Huyut and Kocaturk, 2022; Tahir Huyut et al., 2022). Due to their accessibility and cost-effectiveness, RBT data have been widely used in artificial intelligence studies for disease diagnosis and prognosis (Huyut and Huyut, 2021, 2023; Santos-Silva et al., 2024; Üstündag et al., 2023). Recent clinical research has demonstrated the utility of RBT in the early detection and prognosis of COVID-19 and other diseases (Huyut and Ilkbahar, 2021; Huyut and Velichko, 2023; Mertoglu et al., 2022). For instance, Huyut et al. developed successful AI models using RBT data for the diagnosis and prognosis of COVID-19 (Huyut and Ustundag, 2022; Huyut and Velichko, 2022; Huyut et al., 2022; Velichko et al., 2022). Similarly, Yan et al. (2021) applied the Gradient Boosting Decision Tree method to RBT data for the early diagnosis of multiple myeloma (Yan et al., 2021). Other studies have also highlighted the potential of RBT-based AI models in various clinical contexts. For example, Soerensen et al. (2022) developed an AI model for cancer risk prognosis in primary care using RBT data (Soerensen et al., 2022), while Wu C. C. et al. (2019) achieved 95.7% accuracy in predicting lung cancer using the Random Forest algorithm based on 19 routine blood test parameters (Wu J. et al., 2019). Additionally, Wu J. et al. (2019) utilized the Random Forest method to predict high-risk liver diseases (Wu C. C. et al., 2019), and Kawakami et al. (2019) applied AI models such as Naive Bayes, Artificial Neural Networks, and Elastic Net for the preoperative diagnosis and prognosis of epithelial ovarian cancer (Kawakami et al., 2019). More recently, Haider et al. (2022) used artificial neural networks to analyze RBT data for early differentiation among leukemias (Haider et al., 2022). These studies underscore the significant potential of RBT data in enhancing disease prognosis and management through AI techniques.

Machine learning (ML) techniques, which excel at handling complex and high-dimensional data, have garnered widespread attention for their ability to improve predictive accuracy when used to build prognostic models (Fan et al., 2023; Tian et al., 2023). However, despite the superior performance of ML models, their “black box” (Azodi et al., 2020) nature makes it difficult to interpret the basis of their prognoses, thus limiting their clinical applicability. To address the challenge of model interpretability, the SHapley Additive exPlanation (SHAP) method offers an effective solution. SHAP, based on Shapley values from game theory, quantifies the contribution of each input feature to the model's prognosis, providing interpretability for complex ML models (Mosca et al., 2022; Zhou et al., 2024). SHAP helps visualize how each feature influences the prognosis, enabling clinicians to better understand the decision-making process of ML models, and thus increasing the credibility and usability of these models in clinical practice (Feretzakis et al., 2024). For a disease like SFTS, which is influenced by multiple factors, SHAP can reveal the role of various clinical features in prognosis, facilitating the development of individualized treatment plans.

Despite many previous studies identifying risk factors for death from Thrombocytopenia Syndrome among adults, there is still a need to clarify the characteristics affecting Thrombocytopenia Syndrome. Our study, modeling “comorbidities and laboratory tests” together, provides low-cost, rapid and reliable results on Thrombocytopenia Syndrome. This study aims to develop a machine learning-based prognostic model for SFTS and improve its interpretability using SHAP, ultimately resulting in a practical clinical tool based on the model.

## Methods

### Study population

This study is a retrospective multicenter cohort study conducted in China, aiming to derive and validate a prognostic model for patients with SFTS. The derivation cohort consisted of 292 patients treated at The Second Hospital of Nanjing between January 2022 and December 2023, while the external validation cohort included 104 patients from The First Affiliated Hospital of Wannan Medical College during the same period. Inclusion criteria were: SFTS patients were diagnosed based on the presence of acute fever (with a temperature of 38°C or higher) and platelet count <100 × 10^9^/L), with lab-confirmed SFTS virus (SFTSV) infection by qRT-PCR (Huang et al., 2019; Yu and Morita, 2019), hospitalized adults aged 18 years or older, and those with complete demographic information, laboratory test results, and final outcome data. Exclusion criteria: pregnant patients and cancer patients were excluded from the study.

### Data collection and processing

We collected demographic characteristics, vital signs, and laboratory data at admission from the electronic medical record system. Missing values for categorical variables were imputed using the mode, while missing continuous variables were imputed using the median method (Berkelmans et al., 2022). To minimize the impact of different measurement scales on the model, all continuous variables were standardized. The Sequential Organ Failure Assessment (SOFA), Acute Physiology and Chronic Health Evaluation II (APACHE II) scores, and Prognostic Nutritional Index (PNI) were calculated based on the first examination at admission.

To avoid the impact of missing data on model construction, features with more than 20% missing values were excluded. Twenty-four clinical features were selected as candidate features, including: sex, mechanical ventilation (MV), high flow oxygen therapy (HF), continuous renal replacement therapy (CRRT), underlying disease, consciousness, age, temperature (T), heart rate (HR), mean arterial pressure (MAP), white blood cell count (WBC), neutrophil count (N), lymphocyte count (L), platelet count (PLT), urea (U), total bilirubin (TBil), alanine aminotransferase (ALT), aspartate aminotransferase (AST), albumin (ALB), lactate dehydrogenase (LDH), alkaline phosphatase (ALP), creatinine (Cr), D-dimer, and Prognostic Nutritional Index (PNI).

### Model development and comparison

During the feature selection stage, the Boruta algorithm was used to identify the most predictive features from the 24 clinical features, ranking their importance based on Z-scores. The derivation cohort data was split into a 7:3 ratio for training and internal validation sets to avoid overfitting. We progressively incorporated the selected clinical features into 10 different machine learning models to construct prognostic models, including Logistic Regression (LR), Support Vector Machine (SVM), K-Nearest Neighbors (KNN), Naive Bayes (NB), Decision Tree (DT), Random Forest (RF), Gradient Boosting Decision Tree (GBDT), AdaBoost, Voting Classifier, and Extreme Gradient Boosting (XGBoost).

To optimize model performance, we used GridSearchCV for hyperparameter tuning. Each model underwent 5-fold and 10-fold cross-validation to identify the best combination of hyperparameters. Model stability was further validated using bootstrapping, through which we computed confidence intervals for the performance metrics, thereby enhancing the reliability of the model.

Additionally, to evaluate the prognostic performance of APACHE II and SOFA scores, we constructed three groups of models: (1) a multivariable model based on the 7 selected features, (2) a univariable model based solely on the Acute Physiology and Chronic Health Evaluation score (APACHEII score), and (3) a univariable model based solely on the Sequential Organ Failure Assessment Score (SOFA score). All models were trained and validated using the XGBoost algorithm, and their performance was compared using AUC, accuracy, precision, recall, and F1 score. To further assess model performance based on disease severity, we conducted a stratified analysis using the SOFA score. The patients were divided into two groups: Low-risk group: Patients with a SOFA score ≤ 6; High-risk group: Patients with a SOFA score ≥ 7.

### External validation

To assess the external performance of the model, we conducted external validation using data from 104 SFTS patients treated at The First Affiliated Hospital of Wannan Medical College between January 2022 and December 2023. The inclusion and exclusion criteria for the external validation cohort were consistent with those of the derivation cohort. As the primary goal of the external validation was to evaluate the effectiveness of the prognostic model, only the outcome variables and the clinical features included in the final model were collected for these patients.

### Feature selection and model interpretation

In the feature selection phase, we applied the Boruta algorithm to identify the most predictive features from the initial set of 24 clinical features. The algorithm ranked the importance of each feature based on Z-scores. To address the “black box” issue associated with machine learning models, we employed the SHAP method for model interpretation. SHAP provided both global and local explanations, detailing the contribution of each selected feature to the prognostic prognoses. This approach enabled a clearer understanding of how the model arrived at its prognoses, offering transparency for clinical application.

### Web tool deployment based on the Streamlit framework

To facilitate the clinical application of the final prognostic model, we deployed it in a web application built on the Streamlit framework. Users can input the required feature values into the model and receive real-time prognostic probabilities for individual patients, along with relevant visualizations. This tool aids clinicians in conducting personalized risk assessments and making informed decisions.

### Statistical analysis

All data analyses and model construction were conducted in the Python 3.9.13 environment. Feature selection was performed using the Boruta algorithm, while the Delong test was completed in R 4.3.3. Continuous variables with skewed distributions were presented as medians with interquartile ranges (IQRs) and compared using the Mann-Whitney U test or the Kruskal-Wallis H test. Categorical variables were expressed as frequencies and percentages and compared using the chi-square test or Fisher's exact test. The area under the curve (AUC) was used to evaluate the model's predictive performance, and the optimal threshold was determined by maximizing the Youden index (sensitivity + specificity −1). A two-tailed *P* < 0.05 was considered statistically significant.

## Results

### Patient characteristics

**Figure 1:**
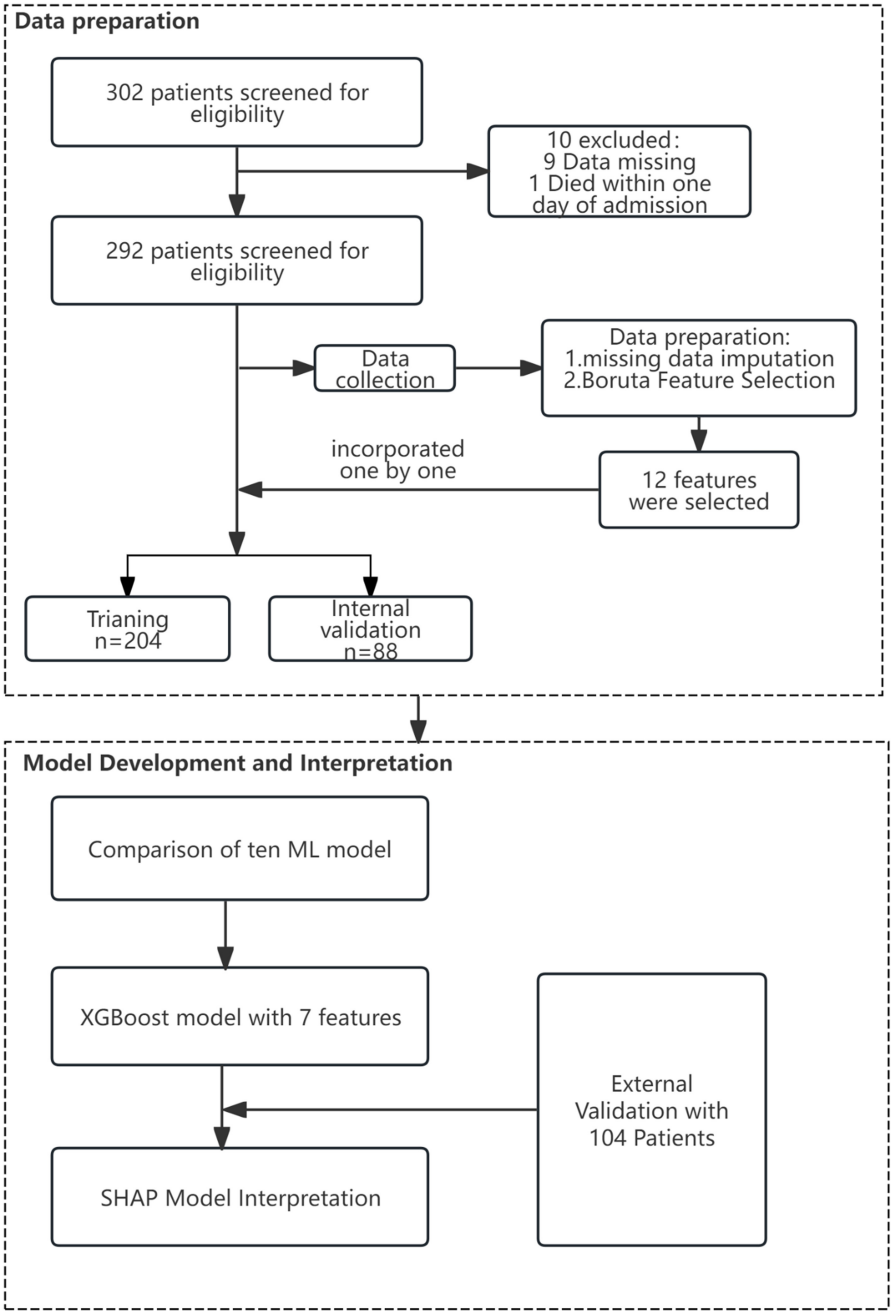
Flow chart of the study design.

This retrospective study included 292 patients from The Second Hospital of Nanjing as the derivation cohort for constructing and validating the prognostic model. Among these 292 patients, 220 survived and 72 died. The mean age of the patients was 65.51 years (standard deviation 10.62, range 25–88 years), with 162 female patients, accounting for 55% of the cohort. The demographic and clinical characteristics of the deceased and surviving groups in the derivation cohort are presented in Table 1. A comparison of demographic and clinical features between the training set, internal validation set, and external validation set can be found in Supplementary Table S1. Details of the study design are displayed in Figure 1.

**Table 1:** Comparison of demographic and clinical characteristics between the survived and died groups.

**Characteristic**	**Survived *N* = 220**	**Died *N* = 72**	***p*-value^a^**
Sex, n (%)			0.28
Female	126 (57%)	36 (50%)	
Male	94 (43%)	36 (50%)	
Age, median [Q1, Q3]	67.00 [56.00, 71.00]	70.00 [65.50, 75.00]	<0.001
APACHEII, median [Q1, Q3]	11.00 [8.00, 16.00]	18.00 [14.00, 23.00]	<0.001
SOFA, median [Q1, Q3]	3.00 [2.00, 4.00]	7.00 [5.00, 10.00]	<0.001
MV, n (%)			<0.001
Not applied	209 (95%)	35 (49%)	
Applied	11 (5.0%)	37 (51%)	
HF, n (%)			0.079
Not applied	214 (97%)	66 (92%)	
Applied	6 (2.7%)	6 (8.3%)	
CRRT, n (%)			<0.001
Not applied	207 (94%)	36 (50%)	
Applied	13 (5.9%)	36 (50%)	
Underlying disease, n (%)			0.22
No comorbidity	119 (54%)	33 (46%)	
Comorbidity	101 (46%)	39 (54%)	
Consciousness, n (%)			<0.001
No change	186 (85%)	15 (21%)	
Changed	34 (15%)	57 (79%)	
T (°C), median [Q1, Q3]	37.90 [36.80, 38.70]	38.50 [37.40, 38.85]	0.005
HR (bpm), median [Q1, Q3]	82.00 [69.50, 90.00]	89.00 [80.00, 104.50]	<0.001
MAP (mmHg), median [Q1, Q3]	82.00 [75.00, 91.00]	84.00 [72.50, 91.50]	0.50
WBC (10^9^/L), median [Q1, Q3]	3.30 [2.07, 5.86]	3.12 [1.87, 4.45]	0.24
N (10^9^/L), median [Q1, Q3]	2.23 [1.07, 4.41]	2.19 [1.15, 3.39]	0.53
L (10^9^/L), median [Q1, Q3]	0.64 [0.43, 1.08]	0.47 [0.31, 1.04]	0.004
PLT (10^9^/L), median [Q1, Q3]	53.00 [36.00, 69.50]	36.50 [27.50, 52.00]	<0.001
U (mmol/L), median [Q1, Q3]	4.95 [3.49, 7.33]	7.62 [5.51, 10.56]	<0.001
TBil (μmol/L), median [Q1, Q3]	7.90 [5.80, 10.70]	9.10 [6.75, 11.75]	0.014
ALT (U/L), median [Q1, Q3]	48.20 [32.35, 80.75]	85.70 [51.05, 146.65]	<0.001
AST (U/L), median [Q1, Q3]	105.20 [60.95, 186.50]	321.20 [194.20, 624.45]	<0.001
ALB (g/L), median [Q1, Q3]	35.30 [32.00, 38.10]	32.80 [30.00, 37.40]	0.007
LDH (U/L), median [Q1, Q3]	493.00 [357.00, 866.50]	1,099.00 [712.00, 1,919.00]	<0.001
ALP (U/L), median [Q1, Q3]	64.00 [48.85, 80.50]	69.00 [55.00, 100.00]	0.020
CR (μmol/L), median [Q1, Q3]	72.10 [59.05, 86.70]	88.50 [71.50, 119.50]	<0.001
D_Dimer (μg/L FEU), median [Q1, Q3]	1.77 [0.83, 4.14]	4.00 [1.78, 13.59]	<0.001
PNI, median [Q1, Q3]	39.33 [36.20, 42.53]	36.95 [33.10, 39.88]	<0.001

a Pearson's Chi-squared test; Wilcoxon rank sum test; Fisher's exact test.

MV, Mechanical Ventilation; HF, High Flow Oxygen Therapy; CRRT, Continuous Renal Replacement Therapy; T, Temperature; HR, Heart Rate; MAP, Mean Arterial Pressure; WBC, White Blood Cell Count; N, Neutrophil Count; L, Lymphocyte Count; PLT, Platelet Count; U, Urea; TBil, Total Bilirubin; ALT, Alanine Aminotransferase; AST, Aspartate Aminotransferase; ALB, Albumin; LDH, Lactate Dehydrogenase; ALP, Alkaline Phosphatase; CR, Creatinine; D-Dimer, D-Dimer; PNI, Prognostic Nutritional Index.

### Model development and performance comparison

**Figure 2:**
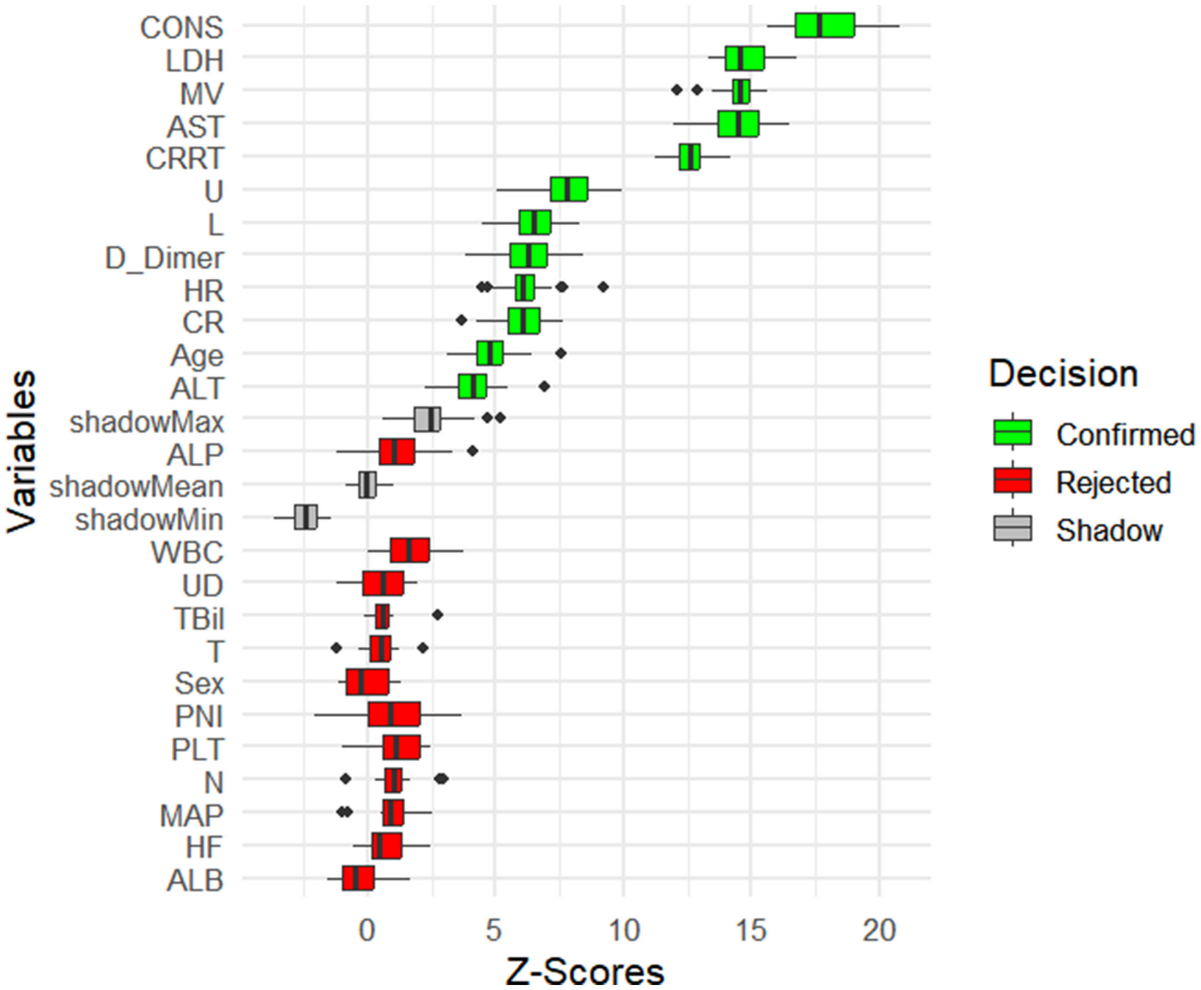
Feature selection using the Boruta algorithm based on Z-scores.

**Figure 3:**
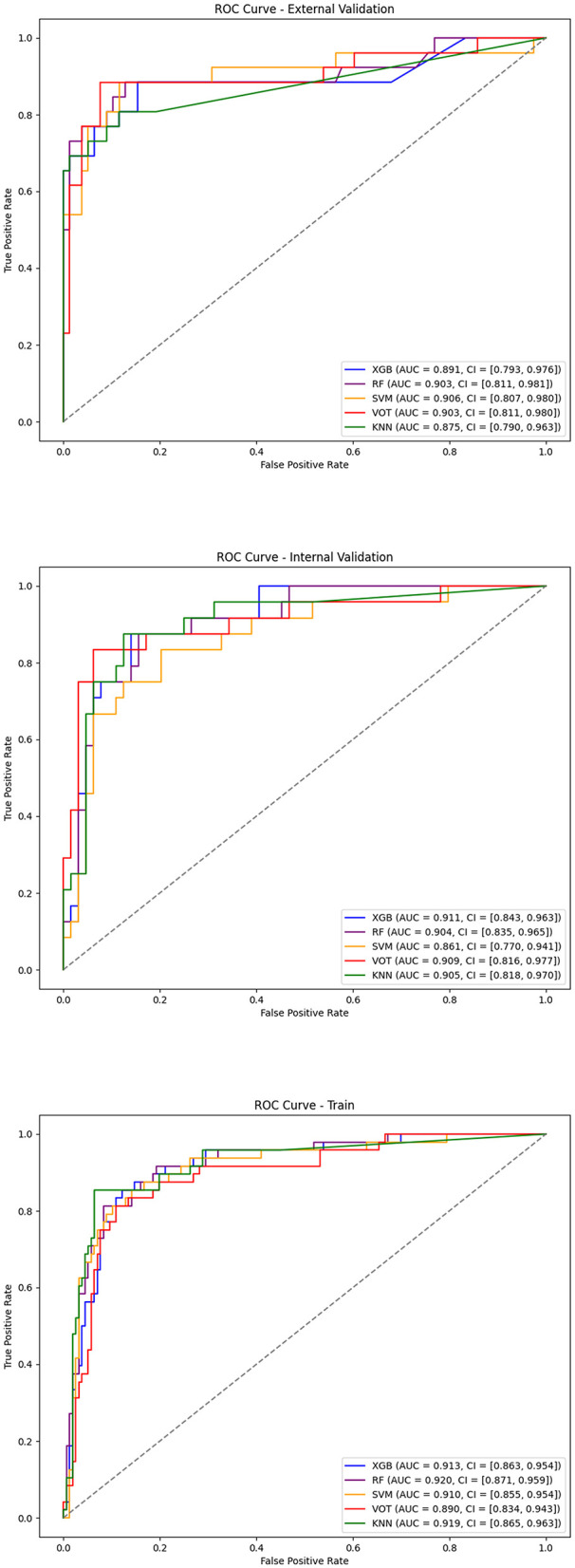
ROC curves of top machine learning models for training, internal validation, and external validation sets.

**Figure 4:**
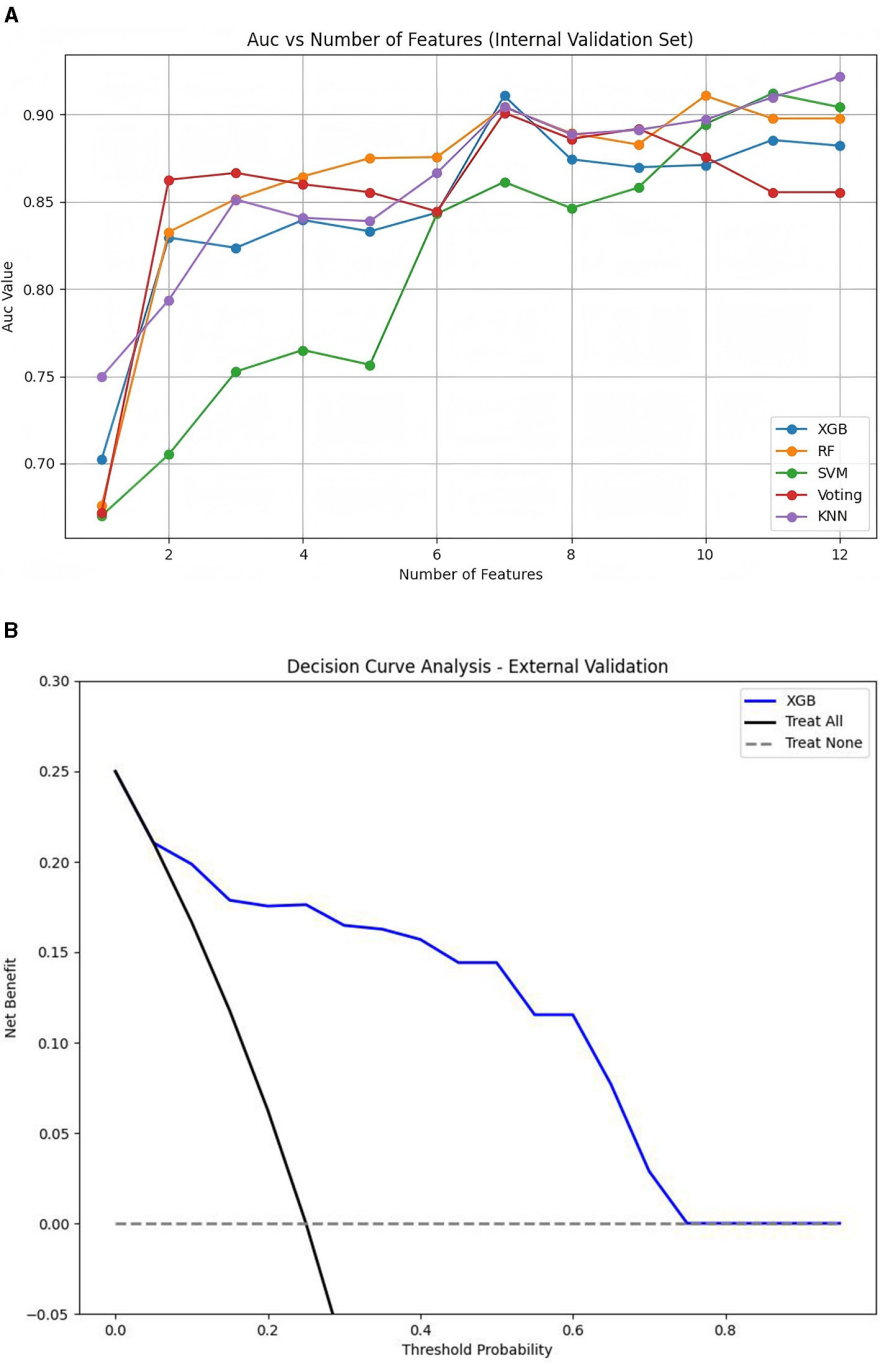
AUC vs. number of features for different models in the internal validation set **(A)** and decision curve analysis (DCA) for the final XGBoost model in the external validation set **(B)**.

First, the Boruta algorithm was used to select 12 of the most predictive features from the initial 24, with their importance ranked based on Z-scores, as shown in Figure 2. Using clinical data collected at patient admission, 10 machine learning models (ML models) were constructed to predict patient prognosis. The primary evaluation metric for this study was AUC, with accuracy, precision, recall, and F1 score used as secondary metrics. The performance of these 10 models is detailed in Supplementary Table S2. Among all models, the top five performers were: KNN (AUC = 0.922, 12 features), SVM (AUC = 0.912, 11 features), RF (AUC = 0.911, 10 features), XGBoost (AUC = 0.911, 7 features), and Voting Classifier (AUC = 0.901, 7 features). The ROC curves for the top five ML models are shown in Figure 3. As the number of included features increased, the AUC showed a general upward trend. For these five models, the performance (AUC) of the Random Forest model with varying numbers of features is shown in Figure 4A, and the other four evaluation metrics are provided in Supplementary Figure S1.

To further optimize the model, the Delong non-parametric test was used to compare models with different numbers of features. For example, the AUC difference between the KNN model (AUC = 0.922, 12 features) and KNN (AUC = 0.905, 7 features) was not significant (*p* = 0.433). Similarly, the difference between the RF model (ΔAUC = 0.007, *p* = 0.653, 7 and 10 features) and the SVM model (ΔAUC = 0.051, *p* = 0.127, 7 and 11 features) was also not statistically significant. Finally, we fixed the number of features at seven, which were: Consciousness, AST, U, CRRT, LDH, L, and MV. Considering the simplicity of the features, we ultimately selected the XGBoost model (AUC = 0.911, seven features) as the final predictive model.

### External validation of the final model

The model performed equally well in the external validation set. The final XGBoost model achieved an AUC of 0.891 (95% CI: 0.786–0.977), an accuracy of 0.894 (95% CI: 0.837–0.952), a precision of 0.907 (95% CI: 0.866–0.955), a recall of 0.894 (95% CI: 0.837–0.952), and an F1 score of 0.884 (95% CI: 0.807–0.949), indicating that the model performed well in both internal and external validations. The DCA curve is shown in Figure 4B.

### Comparison of SOFA and APACHE II scores

The prognostic performance of the SOFA and APACHE II scores was further evaluated and compared with the final selected Seven-feature model. The model incorporating the SOFA score achieved an AUC of 0.872, accuracy of 0.870, precision of 0.815, recall of 0.611, and F1 score of 0.698. In contrast, the model incorporating the APACHE II score showed an AUC of 0.845, accuracy of 0.819, precision of 0.807, recall of 0.347, and F1 score of 0.485. In the low-risk group (SOFA score ≤ 6), the model achieved an AUC of 0.8376 (95% CI: 0.7571–0.9048). In the high-risk group (SOFA score ≥ 7), the AUC was 0.6000 (95% CI: 0.3617–0.8257). These results indicate that the model performed significantly better in the low-risk group compared to the high-risk group.

### Model interpretation

**Figure 5:**
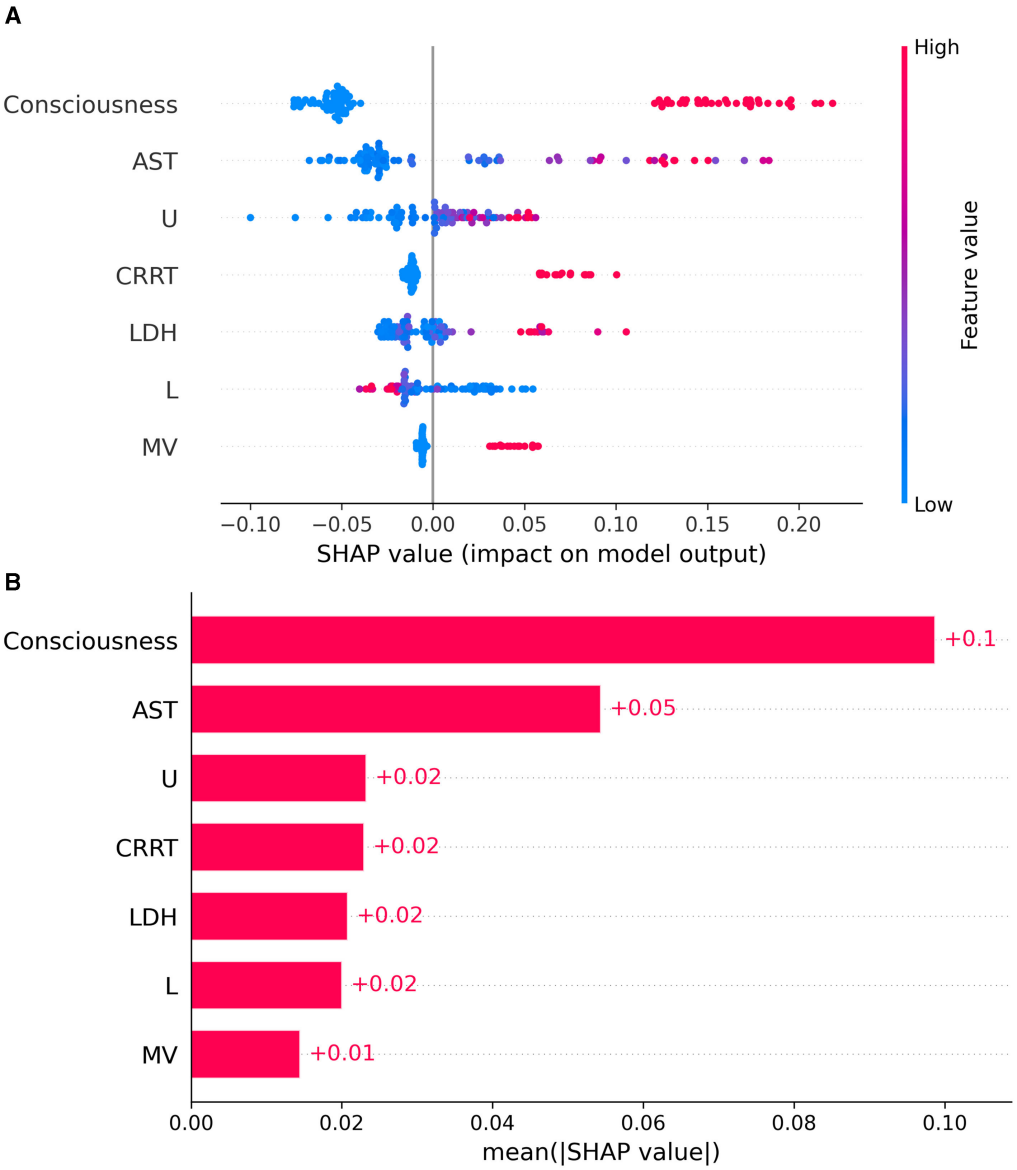
SHAP analysis showing the impact of each feature on model output **(A)** and the mean SHAP values for feature importance **(B)**.

To make the model's prognoses more understandable for clinicians, this study used the SHAP algorithm to explain the importance of each predictor in the XGBoost model's prognoses. At the global interpretation level, the variable importance plot, ranked in descending order of contribution, demonstrated the importance of each variable in the model, as shown in Figures 5A, B. In the model, death was defined as the positive class, and survival as the negative class. The study found that changes in the patient's consciousness at admission were the most predictive of mortality risk, followed by AST, U, CRRT, LDH, L, and MV.

**Figure 6:**
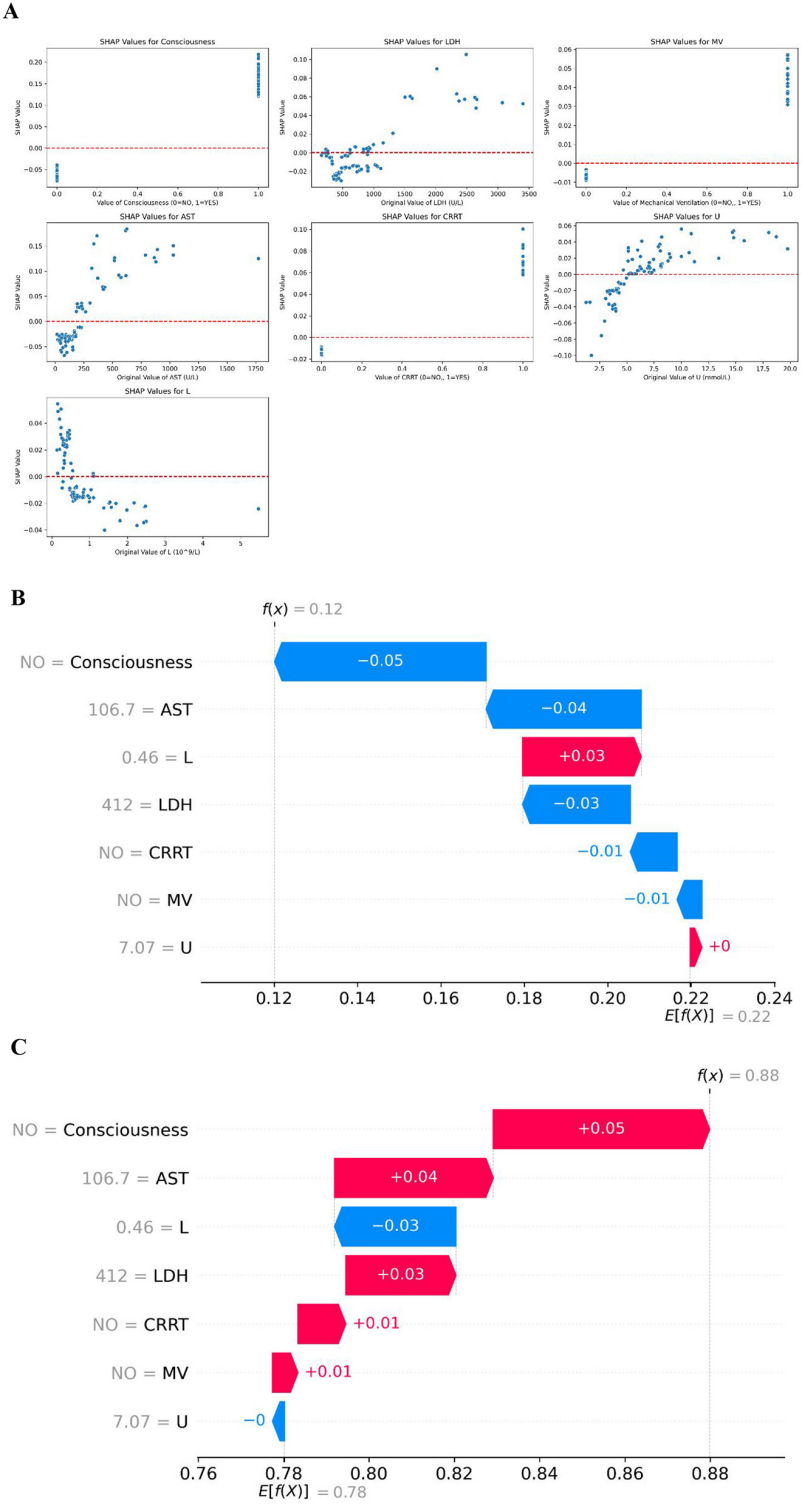
SHAP dependency plots and local explanation for individual prognoses. **(A)** SHAP dependency plots for the seven selected features, illustrating the relationship between feature values and their impact on model output. **(B)** SHAP local explanation for a patient with a predicted 12% probability of death, showing the contribution of each feature to the prognosis. **(C)** SHAP local explanation for a patient with a predicted 88% probability of survival, showing the contribution of each feature to the prognosis.

To further explore the positive and negative correlations of each feature with the prognosis outcomes, SHAP dependency plots were used to show how each feature influences the model's output, with comparisons of the actual values and SHAP values for the seven features, as shown in Figure 6A. In the context of mortality risk prognosis, features with SHAP values greater than zero were associated with an increased probability of the positive class, i.e., a higher risk of death. For instance, if a patient exhibited altered consciousness, the corresponding SHAP value would exceed zero, indicating that this feature contributed to a higher predicted likelihood of mortality. Similarly, urea levels above 4.966 mmol/L were associated with a shift in the model's prognosis toward the “death” category, reflecting an elevated risk of death.

Local explanations provided insights into individual patient prognoses by visualizing the contribution of each feature. As illustrated in Figure 6B, one patient was predicted to have a 12% probability of mortality, placing them in the “death” category. In contrast, Figure 6C demonstrates another patient with an 88% probability of survival. The figures reveal that urea (U) had minimal influence on the prognosis for this patient. Instead, features such as consciousness, AST, LDH, CRRT, and MV contributed to pushing the prognosis toward the “survival” category, while the feature L shifted the prognosis toward the “death” category. These interpretations highlight how changes in specific features can meaningfully alter an individual's predicted probability of survival or death.

### Clinical application of the app

**Figure 7:**
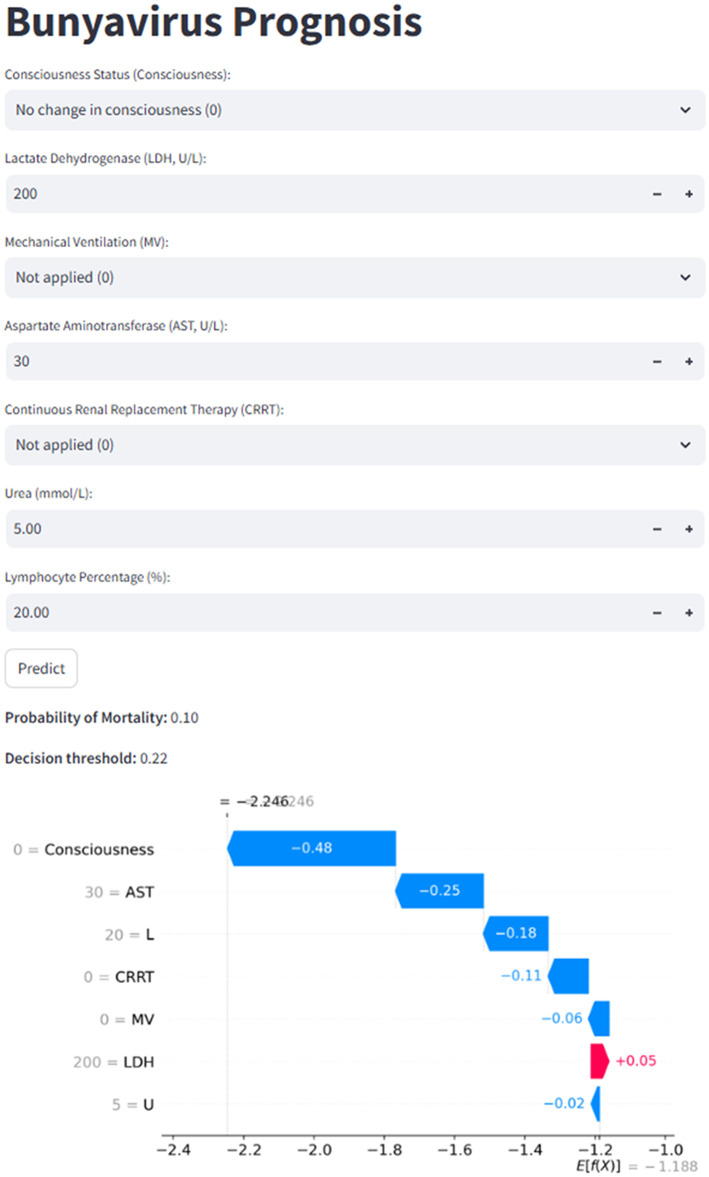
Bunyavirus prognosis: predictive model interface with SHAP-based explanation.

To support clinical use, this study developed a web application based on the Streamlit framework, offering clinicians a tool for real-time, individualized prognostic prognoses (Figure 7). By entering seven key feature values, the app automatically calculates the patient's mortality risk and compares it against a predefined decision threshold. If the predicted probability exceeds the threshold, the patient is classified as being at high risk of death. Additionally, a waterfall plot is provided, with blue bars indicating features that push the outcome toward “survival,” and red bars representing those that push it toward “death.” The web application can be accessed at the following link: https://jft6k52hfhpem8fqrfympd.streamlit.app/.

## Discussion

### Main findings

This study successfully developed an XGBoost-based machine learning model, which demonstrated excellent predictive performance in both internal and external validation cohorts (Internal Validation: AUC = 0.911; External Validation: AUC = 0.891). These findings have significant clinical and theoretical implications. First, the model simplifies complex information by selecting a small number of high-value features, maintaining high accuracy while reducing the difficulty and cost of data acquisition, making it more feasible for clinical application. This is particularly important because existing prognostic models often rely on a large volume of laboratory and imaging data that are not easily accessible, limiting their use in actual clinical practice (Chen et al., 2023; Wang et al., 2024; Zheng et al., 2023; Zhou et al., 2024).

In this study, SOFA and APACHE II scores, which are traditional tools for assessing disease severity (Cheon et al., 2023; Kumar et al., 2020), were widely used in clinical settings and have been proven valuable for prognostic evaluation. In the external validation, the model based on the SOFA score performed well, with an AUC of 0.872, accuracy of 0.870, and precision of 0.815. This indicates that the SOFA score effectively captures disease severity and accurately predicts patient prognosis. However, although the APACHE II score is widely recognized in clinical practice, its performance in this study was relatively inferior, with an AUC of only 0.845, a recall of 0.347, and an F1 score of 0.485. This suggests that the APACHE II score is inadequate for identifying high-risk patients (i.e., positive class samples). In contrast, the final model, which incorporated multiple features, significantly outperformed the single-score models in external validation, achieving an AUC of 0.891 and an F1 score of 0.884, demonstrating superior predictive power and ability to identify positive samples. Therefore, while SOFA and APACHE II scores have clinical value, our multi-feature model provides a more accurate and reliable tool for prognostic assessment of SFTS patients in a multicenter setting.

To further understand the contribution of individual features, SHAP analysis was performed. This analysis revealed the importance of each feature in the model's prognostic predictions, enhancing our understanding of the factors influencing patient outcomes. Among all the features included in the model, changes in consciousness and aspartate aminotransferase (AST) were identified as the most predictive features. AST, traditionally recognized as an indicator of liver dysfunction and systemic inflammatory response, typically reflects multi-organ damage when elevated, particularly in the context of viral infections. However, in our study, elevated AST levels were associated with improved survival, a finding that contrasts with conventional clinical expectations. This paradoxical result may be attributed to the unique characteristics of our study population or the specific disease context. For instance, mild to moderate AST elevation in our cohort might indicate an adaptive metabolic response or a marker of effective treatment rather than severe organ damage. Additionally, the interaction between AST and other clinical features (e.g., early interventions such as antiviral therapy) could have contributed to this observed association. While previous studies have shown that AST levels are closely related to multi-organ failure in SFTS patients, our findings suggest a more nuanced relationship between AST elevation and patient outcomes, highlighting the need for further investigation into the underlying mechanisms (Du et al., 2024; He et al., 2021; Wang et al., 2023). Altered consciousness may be an early signal of central nervous system involvement, indicating that the disease has progressed to a critical stage, aligning with reports in other studies that highlight neurological symptoms as indicators of poor prognosis (He et al., 2021; Xu et al., 2021).

Notably, the Boruta-selected features (e.g., consciousness, AST, CRRT) align with key predictors identified by clinical experts, such as neurological status and organ dysfunction markers. However, the algorithm also highlighted less intuitive features (e.g., urea), which may reflect novel interactions in SFTS pathophysiology. This synergy between data-driven selection and clinical expertise strengthens the model's biological plausibility.

Similarly, the importance of lactate dehydrogenase (LDH) and high-flow oxygen therapy (HF) was validated in the model. LDH is a marker of cell damage and tissue necrosis, and its elevation reflects the extent of viral damage to multiple organs (He et al., 2021; Jia et al., 2017). The application of high-flow oxygen therapy, as a common respiratory support measure, indicates the severity of respiratory system impairment, and this variable showed a significant impact on prognostic prognosis in the model (Jia et al., 2017). Furthermore, the use of continuous renal replacement therapy (CRRT), often seen in patients with renal failure, not only reflected the severity of the disease but also indirectly indicated the level of medical intervention the patient received. This suggests that future model development may benefit from incorporating more features related to therapeutic interventions, which could further enhance the model's predictive ability (Li et al., 2024a).

### Limitations

Despite the multi-level analysis demonstrating the potential of the XGBoost model for SFTS prognosis, several limitations need to be addressed.

First, data source limitations: The data in this study were collected from two centers in eastern China. The epidemiological characteristics and viral strains of SFTS may vary by region, and the model's performance in other areas (such as Japan and South Korea) or different healthcare settings remains unclear. Previous studies have shown that machine learning models may perform differently across regions and ethnic backgrounds, suggesting that future research should include more geographically and ethnically diverse data for validation (Cui et al., 2024; Li et al., 2024b). Additionally, while the model incorporates post-discharge follow-up data to predict long-term outcomes, its current validation focuses on in-hospital mortality and short-term survival. Future studies should explicitly evaluate performance in long-term recovery scenarios.

Second, the “black box” issue of machine learning models: Although SHAP provided some level of explanation for the feature contributions in the XGBoost model, this interpretation still relies on feature importance rankings and does not fully reveal the complex interactions between features. For example, the explanations for CRRT and LDH are primarily focused on their individual effects, without exploring how these features interact with other factors in a multi-feature context (Hong et al., 2022; Manikandan et al., 2024). Therefore, future research could incorporate causal inference models or complex network analysis to further elucidate the interactions and mechanisms between features.

Third, the inherent bias of retrospective studies: As a retrospective cohort study, the electronic medical record data used may contain incomplete or inconsistent records, potentially introducing bias. Recent evidence-based studies suggest that constructing multi-factor confounding models can reduce bias in retrospective analyses, but this approach was not fully adopted in our study (Li et al., 2024b; Manikandan et al., 2024). Additionally, differences in treatment management between hospitals may affect patient outcomes, and this heterogeneity was not fully quantified in the current model (Manikandan et al., 2024). Future research should incorporate more treatment-related information.

Finally, while the model demonstrated real-time applicability with rapid computation (0.5 s per prognosis) and minimal feature requirements, its deployment in clinical settings must adhere to data privacy regulations (e.g., China's PIPL and GDPR). All patient data were anonymized and encrypted during this study, and future implementations will require strict access controls and ongoing compliance audits.

## Conclusion

This study developed an XGBoost-based prognostic model for SFTS, providing both global and local explanations through SHAP, demonstrating efficient and transparent predictive capabilities. The development and validation of the model not only confirmed XGBoost's efficiency and accuracy in handling complex medical data but also highlighted the potential of incorporating interpretability into machine learning models. Although the model performed well on data from two hospitals, further validation of its external generalizability is necessary, especially in different regions and larger sample sizes. Future studies should consider incorporating multi-center, large-scale prospective cohort studies and optimizing the model with more treatment-related features. Additionally, combining complex network analysis and causal inference methods could further reveal the interactions between key features and the pathological mechanisms of the disease, providing a more scientific basis for personalized medicine and targeted interventions.

## Data Availability

The raw data and analysis code can be obtained by contacting the corresponding author, Yi-Shan Zheng, at 284159264@qq.com.
