# Supplementary material for: A multicenter study on developing a prognostic model for severe fever with thrombocytopenia syndrome using machine learning
Source: Front Microbiol. 2025 Mar 19;16:1557922. doi: 10.3389/fmicb.2025.1557922 (PMC11962041; doi:10.3389/fmicb.2025.1557922)
Supplement: Supplementary file 1 [file Table_1.DOCX]

Supplementary Table S1 Comparison of demographic and clinical characteristics among the

training, internal validation, and external validation cohorts

|  | **Groups** | | | |
| --- | --- | --- | --- | --- |
| **Variable** | **Training** (N = 204) | **Internal Validation** (N = 88) | **Derivation** (N = 292) | **External Validation** (N = 104) |
| **Outcome, n (%)** |  |  |  |  |
| Survived | 156 (76%) | 64 (73%) | 220 (75%) | 78 (75%) |
| Died | 48 (24%) | 24 (27%) | 72 (25%) | 26 (25%) |
| **Sex, n (%)** |  |  |  |  |
| Female | 113 (55%) | 49 (56%) | 162 (55%) |  |
| Male | 91 (45%) | 39 (44%) | 130 (45%) |  |
| **Age, Median [Q1, Q3]** | 68.00 [58.50, 72.00] | 66.50 [56.00, 73.00] | 68.00 [58.00, 72.00] |  |
| **APACHEII, Median [Q1, Q3]** | 13.00 [9.00, 18.00] | 13.00 [9.00, 18.00] | 13.00 [9.00, 18.00] |  |
| **SOFA, Median [Q1, Q3]** | 3.00 [2.00, 5.00] | 4.00 [3.00, 6.00] | 3.00 [2.00, 5.00] |  |
| **MV, n (%)** |  |  |  |  |
| Not Applied | 175 (86%) | 69 (78%) | 244 (84%) | 91 (88%) |
| Applied | 29 (14%) | 19 (22%) | 48 (16%) | 13 (13%) |
| **HF, n (%)** |  |  |  |  |
| Not Applied | 200 (98%) | 80 (91%) | 280 (96%) |  |
| Applied | 4 (2.0%) | 8 (9.1%) | 12 (4.1%) |  |
| **CRRT, n (%)** |  |  |  |  |
| Not Applied | 171 (84%) | 72 (82%) | 243 (83%) |  |
| Applied | 33 (16%) | 16 (18%) | 49 (17%) |  |
| **Underlying Disease, n (%)** |  |  |  |  |
| No Comorbidity | 101 (50%) | 51 (58%) | 152 (52%) |  |
| Comorbidity | 103 (50%) | 37 (42%) | 140 (48%) |  |
| **Consciousness, n (%)** |  |  |  |  |
| No Change | 149 (73%) | 52 (59%) | 201 (69%) | 85 (82%) |
| Changed | 55 (27%) | 36 (41%) | 91 (31%) | 19 (18%) |
| **T (°C), Median [Q1, Q3]** | 38.00 [36.80, 38.70] | 38.05 [37.00, 38.80] | 38.00 [36.80, 38.75] |  |
| **HR (bpm), Median [Q1, Q3]** | 84.00 [72.00, 92.00] | 85.00 [74.00, 98.50] | 84.50 [74.00, 94.00] |  |
| **MAP (mmHg), Median [Q1, Q3]** | 83.50 [74.00, 91.00] | 82.00 [75.00, 94.50] | 83.00 [75.00, 91.00] |  |
| **WBC (10^9/L), Median [Q1, Q3]** | 3.45 [2.09, 5.14] | 2.75 [1.58, 5.80] | 3.16 [2.00, 5.31] |  |
| **N (10^9/L), Median [Q1, Q3]** | 2.38 [1.15, 4.14] | 1.79 [1.00, 4.14] | 2.21 [1.09, 4.14] |  |
| **L (10^9/L), Median [Q1, Q3]** | 0.65 [0.42, 1.09] | 0.55 [0.36, 0.92] | 0.61 [0.40, 1.08] | 0.70 [0.40, 1.15] |
| **PLT (10^9/L), Median [Q1, Q3]** | 49.50 [34.50, 67.00] | 42.50 [30.50, 62.50] | 47.50 [33.00, 66.00] |  |
| **U (mmol/L), Median [Q1, Q3]** | 5.34 [3.72, 7.98] | 5.80 [4.03, 8.09] | 5.55 [3.78, 8.06] | 6.19 [4.70, 9.35] |
| **TBil (μmol/L), Median [Q1, Q3]** | 8.10 [6.00, 11.20] | 7.85 [5.80, 10.70] | 8.00 [5.90, 11.10] |  |
| **ALT (U/L), Median [Q1, Q3]** | 57.45 [35.90, 92.20] | 51.15 [33.15, 95.90] | 55.90 [35.85, 92.80] |  |
| **AST (U/L), Median [Q1, Q3]** | 132.00 [70.85, 259.75] | 141.30 [90.05, 284.15] | 135.00 [75.40, 262.25] | 136.00 [82.50, 265.00] |
| **ALB (g/L), Median [Q1, Q3]** | 34.95 [31.55, 38.05] | 34.50 [31.85, 37.20] | 34.90 [31.60, 37.95] |  |
| **LDH (U/L), Median [Q1, Q3]** | 577.00 [365.50, 983.00] | 684.50 [413.00, 931.00] | 613.00 [376.00, 961.00] | 550.50 [388.50, 1,002.50] |
| **ALP (U/L), Median [Q1, Q3]** | 65.50 [49.50, 82.50] | 66.00 [49.00, 87.50] | 66.00 [49.00, 84.00] |  |
| **CR (μmol/L), Median [Q1, Q3]** | 74.95 [61.00, 93.50] | 76.25 [65.75, 91.90] | 75.00 [62.50, 93.00] |  |
| **D_Dimer (μg/L FEU), Median [Q1, Q3]** | 2.19 [0.97, 5.30] | 1.99 [0.85, 4.17] | 2.17 [0.95, 4.91] |  |
| **PNI, Median [Q1, Q3]** | 38.78 [35.55, 41.88] | 38.10 [34.55, 41.40] | 38.58 [35.20, 41.83] |  |

Abbreviations: MV (Mechanical Ventilation), HF (High Flow Oxygen Therapy), CRRT (Continuous Renal Replacement Therapy), T (Temperature), HR (Heart Rate), MAP (Mean Arterial Pressure), WBC (White Blood Cell Count), N (Neutrophil Count), L (Lymphocyte Count), PLT (Platelet Count), U (Urea), TBil (Total Bilirubin), ALT (Alanine Aminotransferase), AST (Aspartate Aminotransferase), ALB (Albumin), LDH (Lactate Dehydrogenase), ALP (Alkaline Phosphatase), CR (Creatinine), D-Dimer (D-Dimer), PNI (Prognostic Nutritional Index).


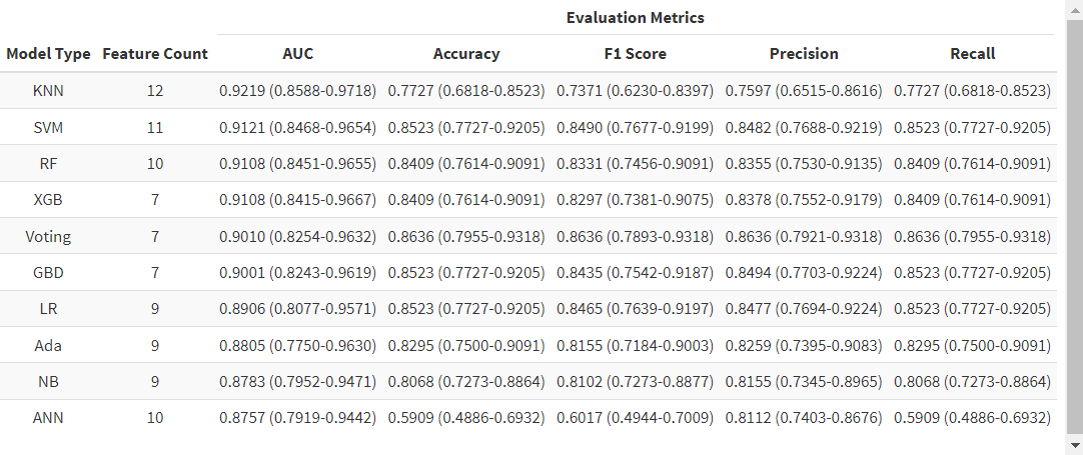


Supplementary Table S2 Performance Comparison of Machine Learning Models Using Various Evaluation Metrics

A
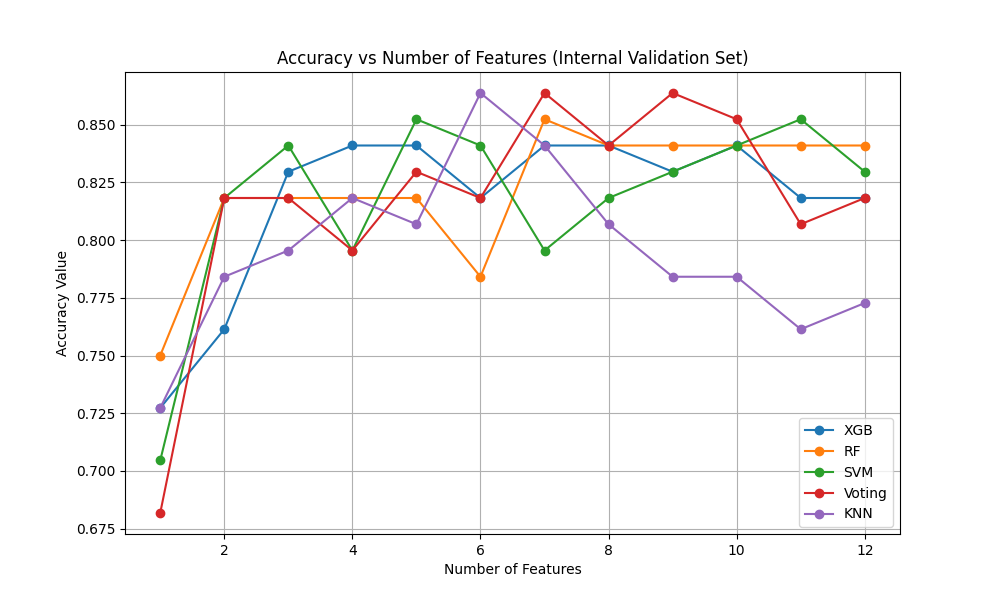


B


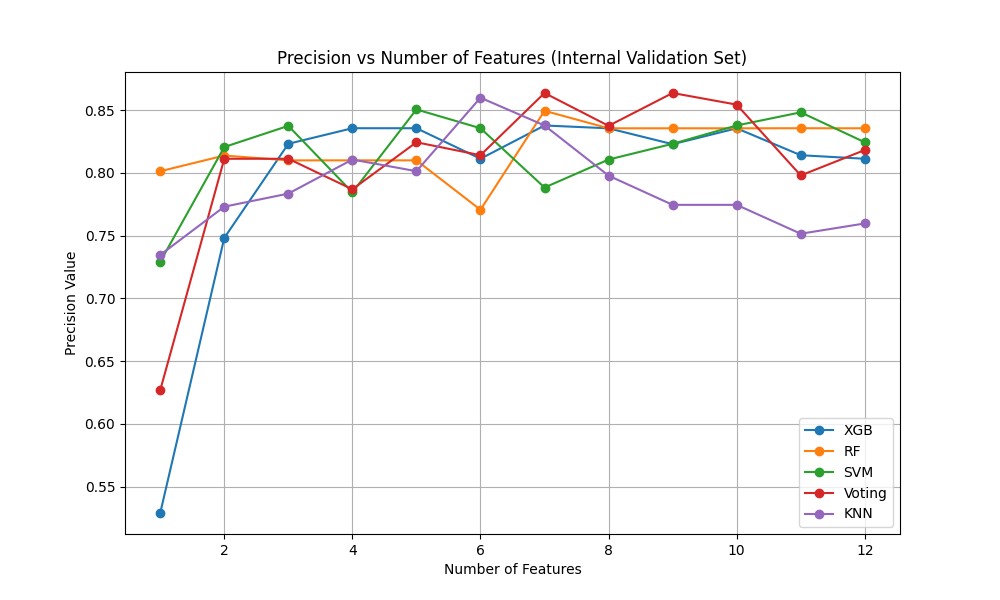


C


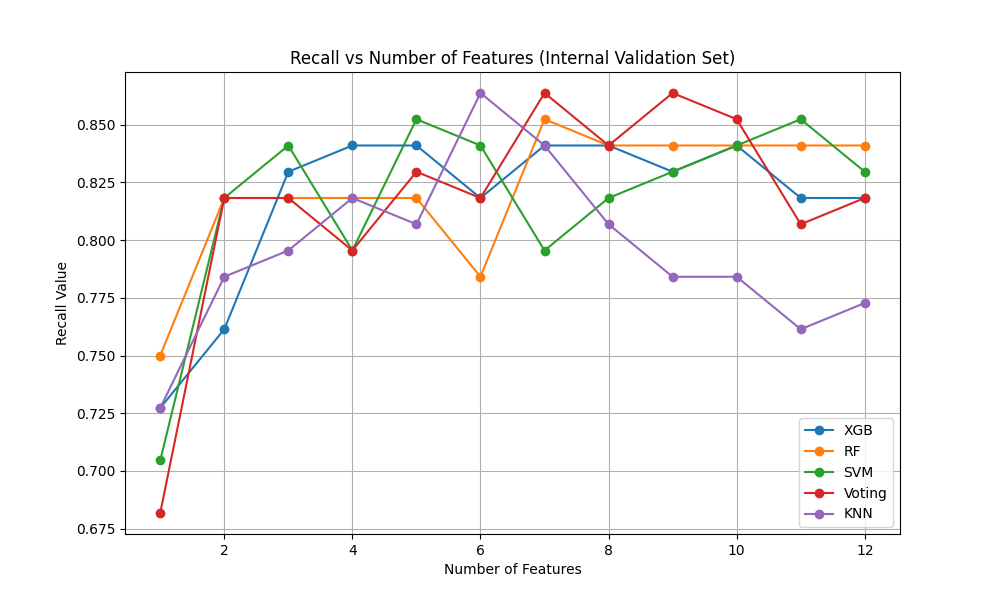


D


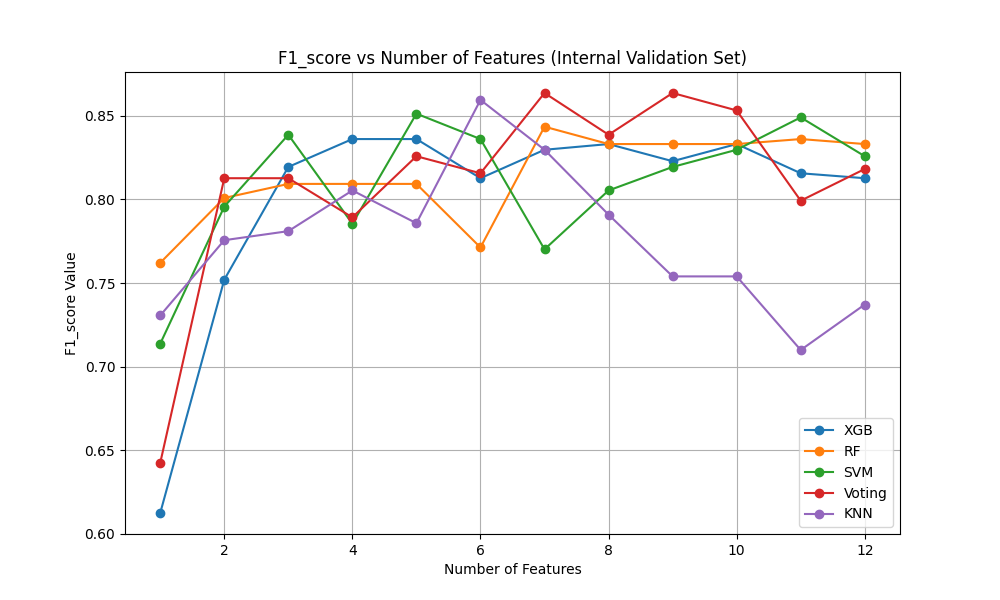


Supplementary Fig. S1: Relationship Between Feature Count and Four Performance Metrics (Accuracy, Precision, Recall, and F1 Score) in the Internal Validation Set
